# Notes on two crabs (Crustacea, Brachyura, Dynomenidae and Iphiculidae) collected from red coral beds in northern Taiwan, including a new species of *Pariphiculus* Alcock, 1896

**DOI:** 10.3897/zookeys.694.14871

**Published:** 2017-08-29

**Authors:** Peter K. L. Ng, M.-S. Jeng

**Affiliations:** 1 Lee Kong Chian Natural History Museum, Faculty of Science, National University of Singapore, 2 Conservatory Drive, Singapore 117377, Republic of Singapore; 2 Biodiversity Research Center, Academia Sinica, 128 Academia Road, Section 2, Nankang, Taipei 11529, Taiwan, R.O.C.

**Keywords:** Brachyuran crab, Dromioidea, East China Sea, Leucosioidea, new *Pariphiculus* species, taxonomy

## Abstract

Two brachyuran species of the families Dynonemidae and Iphiculidae are reported from red coral beds in northern Taiwan. The dynonemid *Acanthodromia margarita* (Alcock, 1899) has hitherto been reported from the Andaman Sea, Japan, and Philippines and the species is here recorded for the first time from Taiwan. A new species of iphiculid, *Pariphiculus stellatus* **sp. n.**, is also described. The new *Pariphiculus*, which also occurs in the Philippines, is superficially similar to *P. agariciferus* Ihle, 1918, a species known from Indonesia, Japan, Philippines, South China Sea, Taiwan, and Vanuatu, but can be distinguished by distinct carapace, pleonal and male first gonopod features.

## Introduction

The small seamount associated with Peng-Chia-Yu Island, a small outcrop 60 km northeast of Keelung in Taiwan, is a special area with controlled commercial fishing for precious red corals (MOJ 2014). One endemic new species, *Corallium carusrubrum* Tu, Dai & Jeng, 2012 (Anthozoa: Octocorallia: Coralliidae) was recently described from this site.

Recently, two crab species were obtained from the area where the precious red corals are collected. One is the rarely reported dromioid *Acanthodromia margarita* (Alcock, 1899) (Dynomenidae), hitherto reported from India, Japan, and Philippines. The second is a new species of leucosioid of the genus *Pariphiculus* Alcock, 1896 (Iphiculidae), which is superficially similar to *P. agariciferus* Ihle, 1918, from Indonesia, Japan, Philippines, South China Sea, Taiwan, and Vanuatu. The taxonomy of these two species is discussed. The new species of *Pariphiculus*, here named *P. stellatus*, is described and compared at length with *P. agariciferus*. *Pariphiculus stellatus* sp. n. is also reported from the Philippines.

## Materials and methods

The measurements provided (in millimetres) are of the maximum carapace width and length (including spines), respectively. The abbreviations G1 and G2 are used for the male first and second gonopods, respectively.

Specimens examined are deposited in the collections of the Institute of Zoology, Biodiversity Research Center, Academia Sinica (**ASIZ**), Taipei, Taiwan; Muséum national d'Histoire naturelle (**MNHN**), Paris, France; and the Zoological Reference Collection (**ZRC**) of the Lee Kong Chian Natural History Museum, National University of Singapore. The terminology used follows that in Tan and Ng (1995) and Davie et al. (2015).

## Systematics

### Family Dynomenidae Ortmann, 1892

#### Genus *Acanthodromia* A. Milne-Edwards, 1880

##### Acanthodromia margarita

Taxon classificationAnimaliaCrustaceaDynomenidae

(Alcock, 1899)

Figs 1A–C , 2 , 3

Dynomene margarita Alcock, 1899: 19, pl. 2: fig. 3.
Acanthodromia margarita – Alcock 1900: 134; Sakai 1965: 43; Sakai 1976: 31, pl. 7: fig. 2; Nagai 1989: 43; McLay 1999: 539, fig. 31; Marumura and Kosaka 2003: 20; McLay and Ng 2005: 18, fig. 5; Ng et al. 2008: 37.

###### Material examined.

**Taiwan**: 1 female (17.8 × 18.3 mm) (ASIZ 75484), waters of Zone A (precious coral fishing ground), Peng-Chia-Yu Island, 60 km northeast of Keelung, 25°37.901'N, 122°28.577'E, 175 m, Taiwan, coll. Fishing Vessel “De-Cheng 136”, M.-L. Chang, 14 May 2017. **Philippines**: 1 male (14.7 × 17.7 mm) (ZRC 2001.358), Balicasag Island, 200–300 m, coll. December 2000; 1 male (10.5 × 11.5 mm), 2 females (7.8 × 8.3 mm, 11.2 × 12.2 mm), 2 ovigerous females (14.7 × 16.7 mm, 17.0 × 18.5 mm) (ZRC 2003.668), 1 male (9.9 × 10.6 mm), 1 female (12.3 × 13.4 mm) (MNHN); Balicasag Island, 200 –300 m, 25 –30 Jul 2003; 1 male (14.7 × 15.8 mm) (ZRC 2008.1425), station PN1, Balicasag Island, 200–300 m, coll. November 2003; 1 female (no pereopods left, 11.2 × 12.0 mm) (ZRC 2008.1426), Balicasag Island, 200–300m, coll. November 2003–April 2004; 1 male (15.3 × 16.7 mm) (ZRC 2008.1419), station PN1, Balicasag Island, coll. 29 May 2004; 1 female (17.7 × 19.2 mm) (ZRC 2008.1420), Balicasag Island, coll. 29 March 2004; 5 males (8.8 × 10.6 mm, 11.2 × 12.5 mm, 11.3 × 13.0 mm, 13.5 × 14.2 mm, 14.1 × 16.2 mm), 2 females (15.4 × 17.2 mm, 15.8 × 17.0 mm), 1 female (carapace cracked) (ZRC 2004.596), Balicasag Island, coll. 2 March 2004; 1 male (11.3 × 12.7 mm) (ZRC 2008.1043), Balicasag Island, coll. May 2004 [all above locations at 9.518891°N, 123.680511°E, Panglao, Bohol, Visayas, Philippines; purchased from local shell fishermen, obtained by tangle nets]; 1 male (13.9 × 15.3 mm), 2 females (14.2 × 15.0 mm, 15.7 × 16.1 mm) (ZRC 2013.372), Maribojoc Bay, Panglao, Bohol, Philippines, coll. J. Arbasto, 30 May 2004; 1 female (14.2 × 15.0 mm) (ZRC 2007.207), Maribojoc Bay, Panglao, Bohol, Philippines, coll. J. Arbasto, July-May 2005.

###### Description.

**Figure 1.**
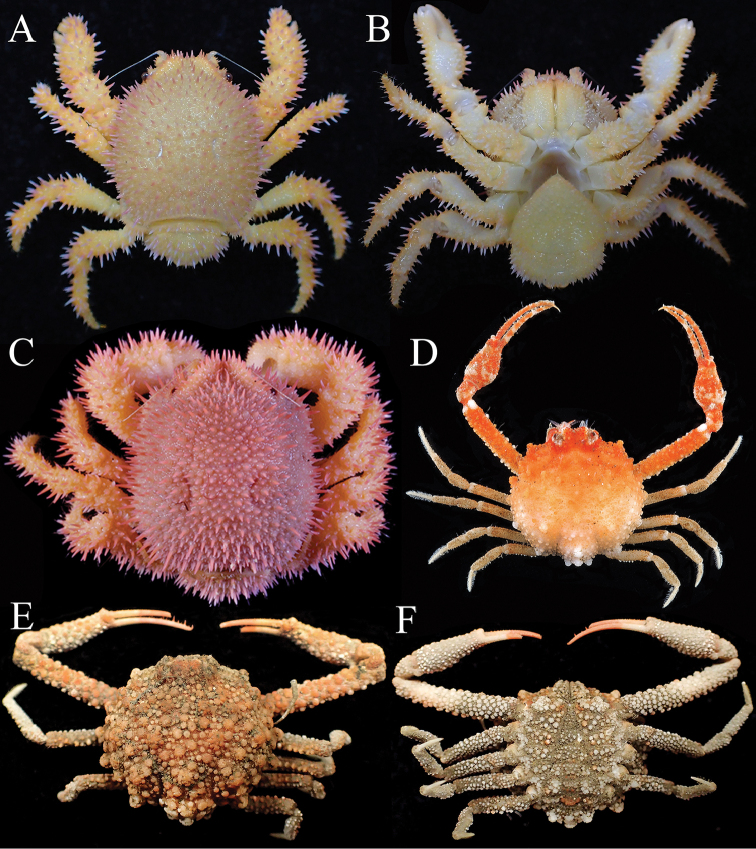
Colour in life. **A, B** *Acanthodromia margarita* (Alcock, 1899), female (17.8 × 18.3 mm) (ASIZ 75484), Taiwan **C** *Acanthodromia margarita* (Alcock, 1899), female (17.7 × 19.2 mm) (ZRC 2008.1420), Philippines **D** *Pariphiculus agariciferus*, Ihle, 1918, male (11.9 × 12.1 mm) (ZRC 2009.471), Vanuatu **E, F** *Pariphiculus stellatus* sp. n., holotype male (27.7 × 24.5 mm) (ASIZ 75485), Taiwan. **A, C, D, E** overall dorsal view; **B, F** ventral view.

*Colour*. McLay and Ng (2005: 18) described the fresh colour of Philippine specimens as “light pink spines on carapace and the pereopods with golden yellow dactyli”. The present Taiwan specimen is orange-yellow on all its dorsal surfaces, with the spines orange-pink with white tips (Fig. 1A, B). In some Philippine specimens, the carapace can be a more striking pink and the pereopods orange-yellow with the spines pink (Fig. 1C).

###### Remarks.

**Figure 2.**
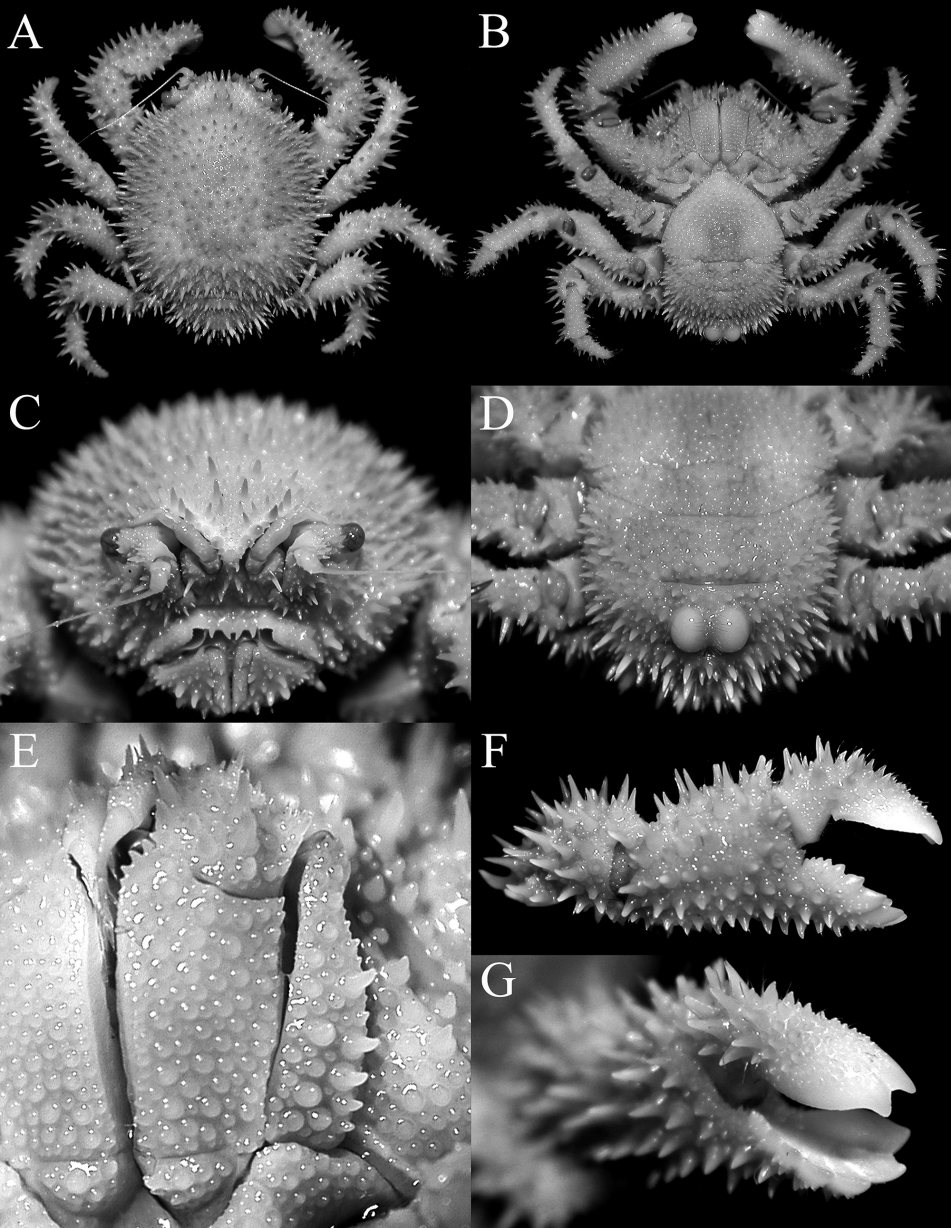
*Acanthodromia margarita* (Alcock, 1899), female (17.8 × 18.3 mm) (ASIZ 75484), Taiwan. **A** overall dorsal view **B** ventral view of cephalothorax **C** frontal view of cephalothorax **D** pleon showing tubercles on somite 4 **E** outer view of left third maxilliped **F** outer view of right chela **G** sublateral view of fingers of right chela.

**Figure 3.**
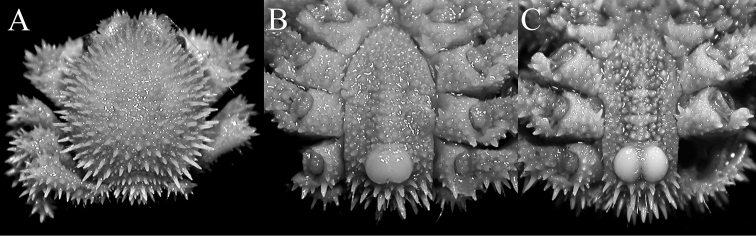
*Acanthodromia margarita* (Alcock, 1899). **A**, **B** male (11.6 × 12.0 mm) (ZRC 2003.668), Philippines **C**, male (13.5 × 14.2 mm) (ZRC 2004.596), Philippines. **A** overall dorsal view **B, C** pleon showing tubercles on somite 4.

The present female specimen from Taiwan is one of the largest on record and agrees well with published descriptions and figures of the species. The frontal margin appears to vary in form due to the relative strength of the frontal spines, particularly the median pseudorostral one. The two large rounded, basally fused tubercles on pleonal somite 4 is distinct in both sexes; with those in some of the smaller specimens appearing almost completely fused, forming one structure (Fig. 3B). The posterior margin of the epistome always has four spines, but the structure of the two median ones varies slightly, from directly pointing downwards (e.g., McLay and Ng 2005: fig. 5B) to curving medially (Fig. 2C).

###### Distribution and depth.

*Acanthodromia margarita* was described from the Andaman Sea in the eastern Indian Ocean, and has been also reported from Japan, Philippines, and now Taiwan. The Indian Ocean specimen was from relatively shallow water (135 m), but the series from the Philippines was from 120–300 m depth. The present Taiwan specimen was collected from a depth of 175 m.

**Figure 4.**
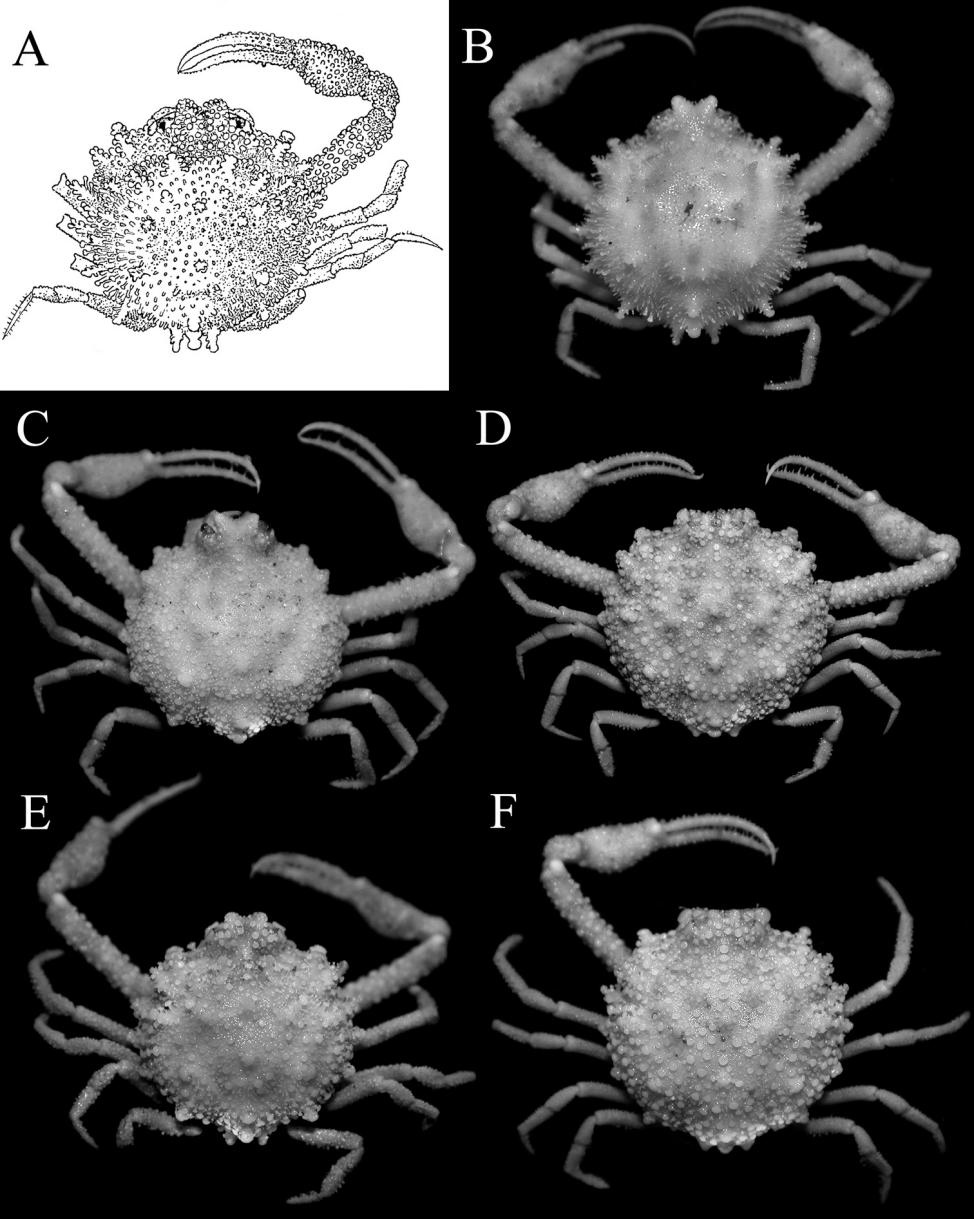
*Pariphiculus agariciferus* Ihle, 1918, overall dorsal view. **A** holotype male (9.0 × 9.3 mm), after Ihle (1918: fig. 230) **B** young male (10.2 × 9.6 mm) (ZRC 2009.470), Vanuatu **C** male (11.9 × 12.1 mm) (ZRC 2009.471), Vanuatu **D** female (22.1 × 21.3 mm) (ZRC 2009.288), Philippines **E** male (15.5 × 14.4 mm) (ZRC 2012.484), Philippines **F** female (19.0 × 19.0 mm) (ZRC 2017.186), Philippines.

**Figure 5.**
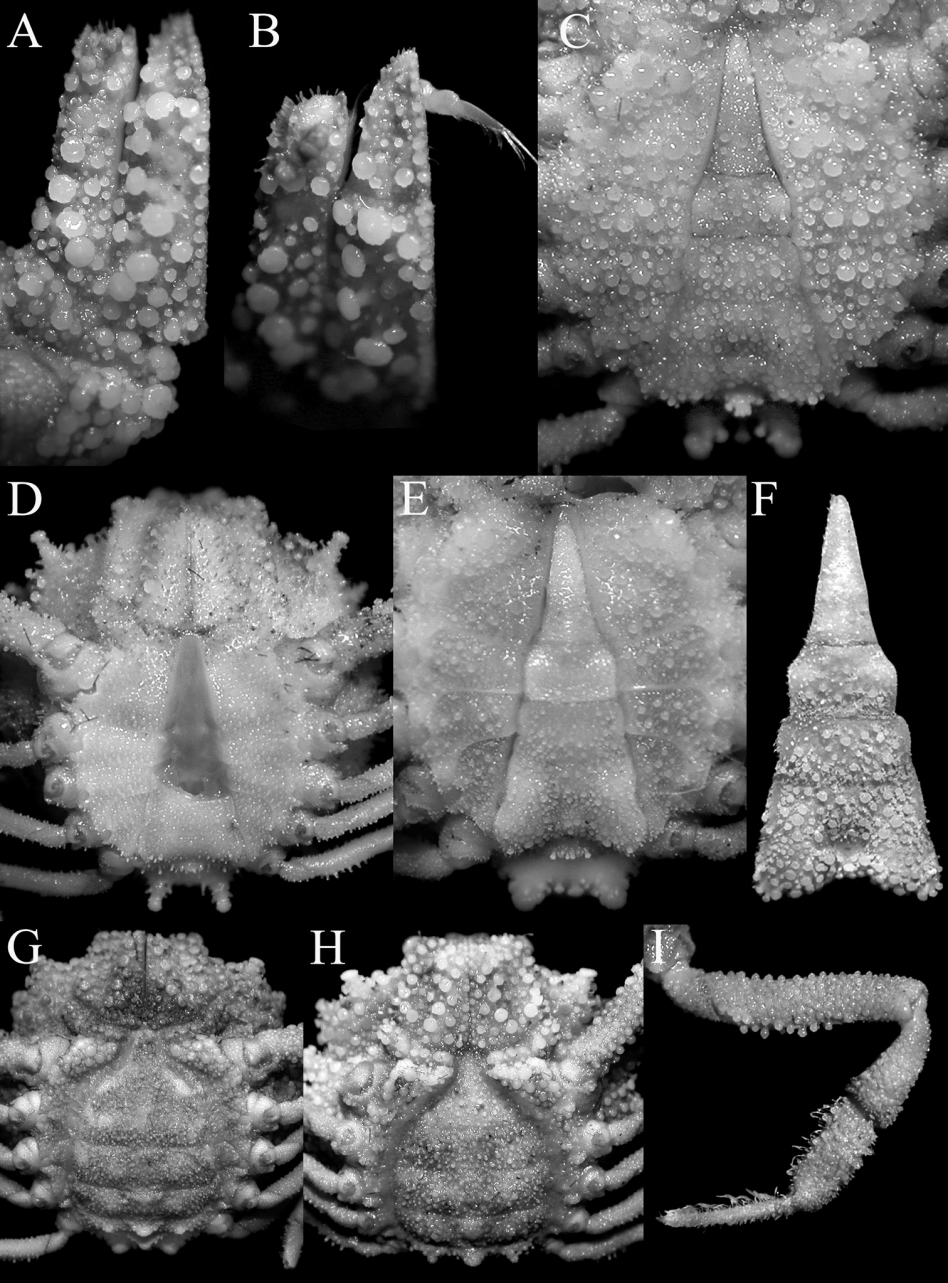
*Pariphiculus agariciferus* Ihle, 1918. **A**, **B** female (19.8 × 18.9 mm) (ZRC 2017.181), Philippines **C** male (15.5 × 14.4 mm) (ZRC 2012.484), Philippines **D** young male (10.2 × 9.6 mm) (ZRC 2009.470), Vanuatu **E** male (11.9 × 12.1 mm) (ZRC 2009.471), Vanuatu **F** male (15.8 × 15.0 mm) (ZRC 2017.187), Philippines **G, I** female (22.1 × 21.3 mm) (ZRC 2009.288), Philippines **H** female (19.0 × 19.0 mm) (ZRC 2017.186), Philippines. **A, B** outer view of right third maxilliped **C–E** male thoracic sternum and pleon **E** female thoracic sternum and pleon **G, H** female thoracic sternum and pleon **E** female thoracic sternum and pleon **I** right fourth ambulatory leg.

**Figure 6.**
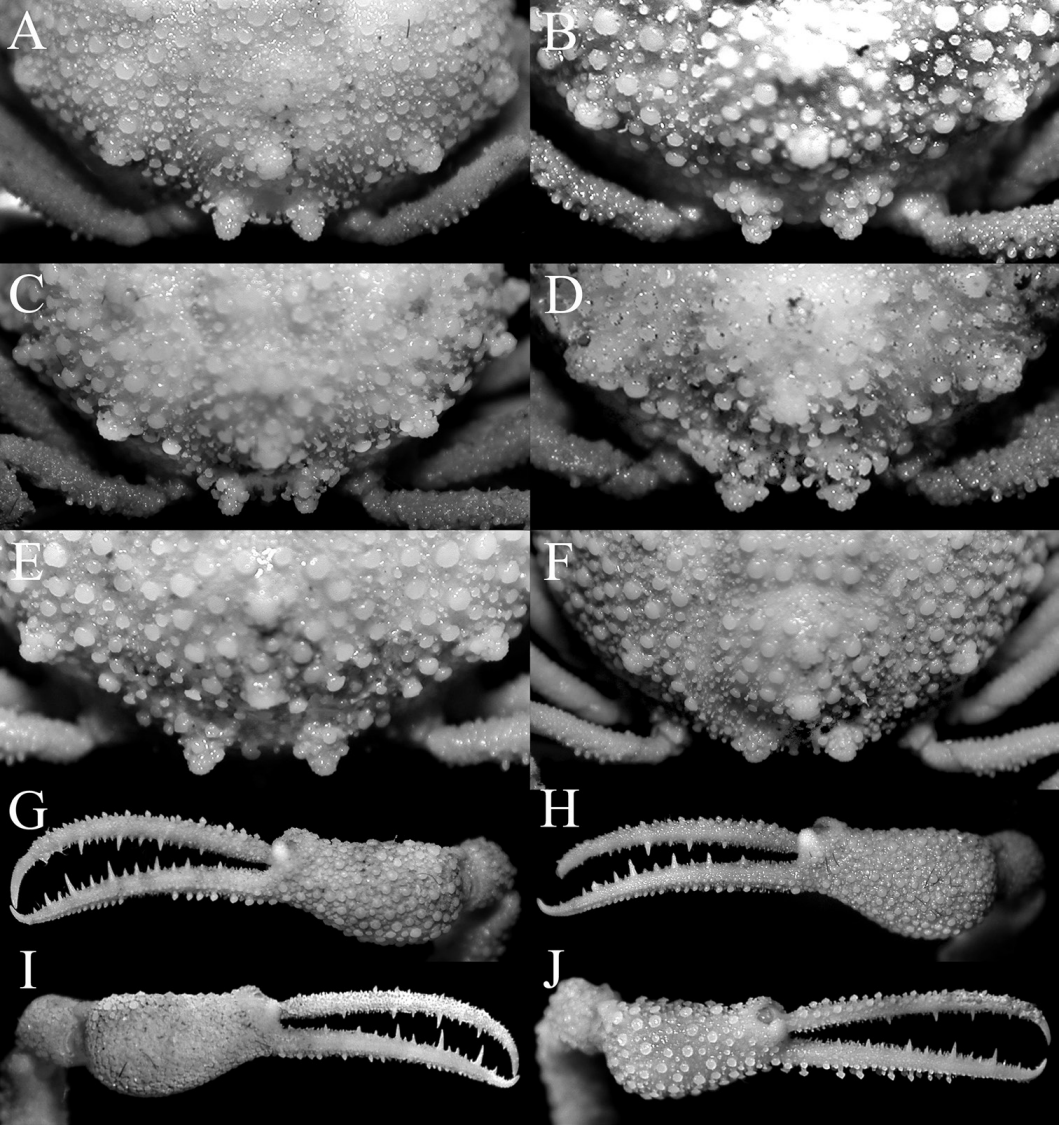
*Pariphiculus agariciferus* Ihle, 1918. **A** male (11.9 × 12.1 mm) (ZRC 2009.471), Vanuatu **B** male (15.8 × 15.0 mm) (ZRC 2017.187), Philippines **C, J** male (15.5 × 14.4 mm) (ZRC 2012.484), Philippines **D** male (12.8 × 13.7 mm) (ZRC 2017.186), Philippines **E, G** female (19.0 × 19.0 mm) (ZRC 2017.186), Philippines **F, H** female (22.1 × 21.3 mm) (ZRC 2009.288), Philippines **I** male (11.9 × 12.1 mm) (ZRC 2009.471), Vanuatu. **A–F** intestinal region and posterior margin of carapace **G, H** outer view of left chela **I**, **J** outer view of right chela.

### Family IPHICULIDAE Alcock, 1896

#### *Pariphiculus* Alcock, 1896

##### Pariphiculus stellatus sp. n.

Taxon classificationAnimaliaCrustaceaIphiculidae

http://zoobank.org/7EC3869E-1F27-4C50-8EE3-DCEB4817630C

**Figure 7.**
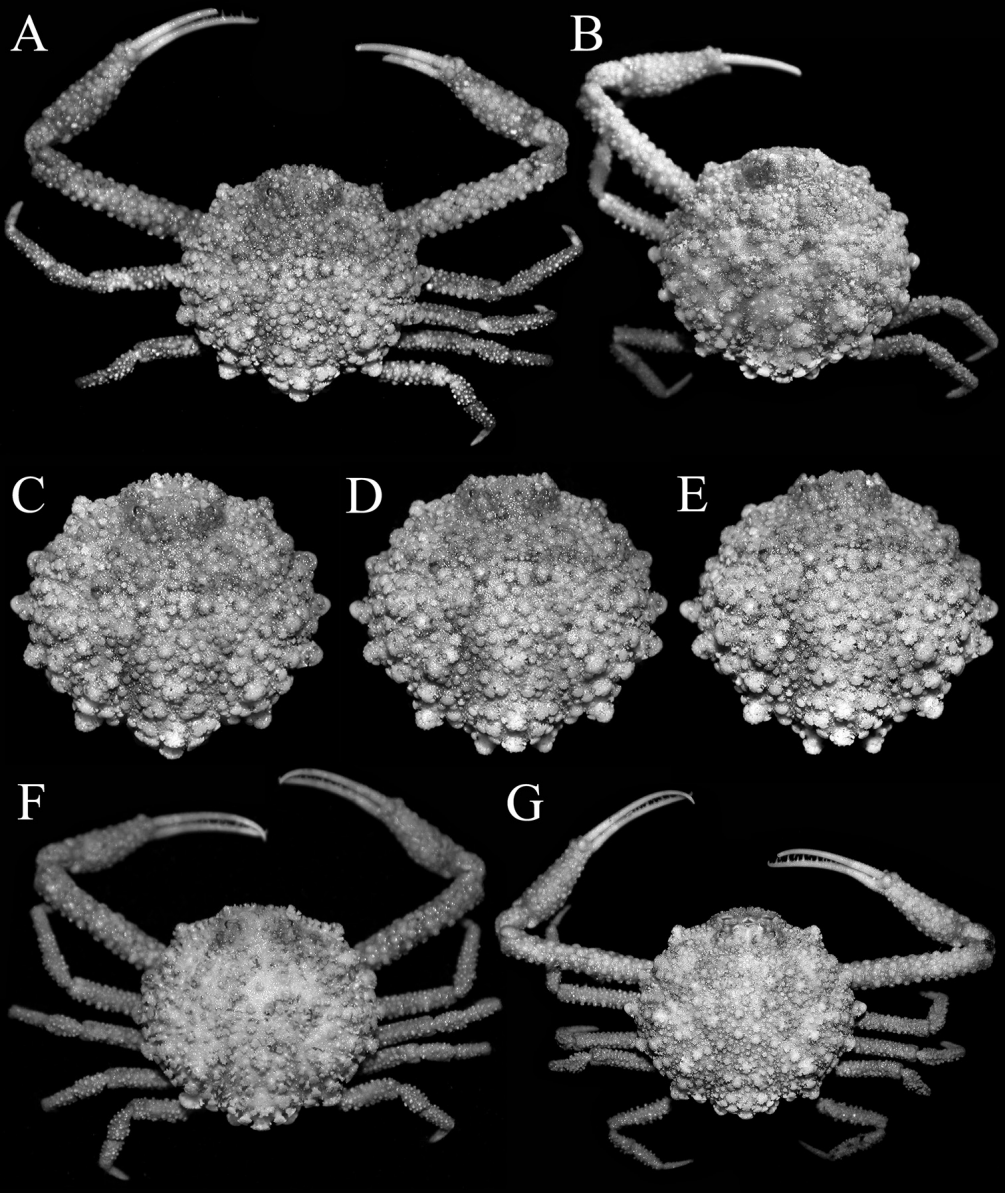
*Pariphiculus stellatus* sp. n. **A, C–E** holotype male (27.7 × 24.5 mm) (ASIZ 75485), Taiwan **B** female (32.3 × 27.6 mm) (ZRC 2017.185), Philippines **F** male (24.3 × 21.6 mm) (ZRC 2007.590), Philippines **G** female (35.1 × 29.6 mm) (ZRC 2007.590), Philippines. **A, B, F, G** overall dorsal view **C–E** dorsal view of carapace photographed from slightly different angles.

**Figure 8.**
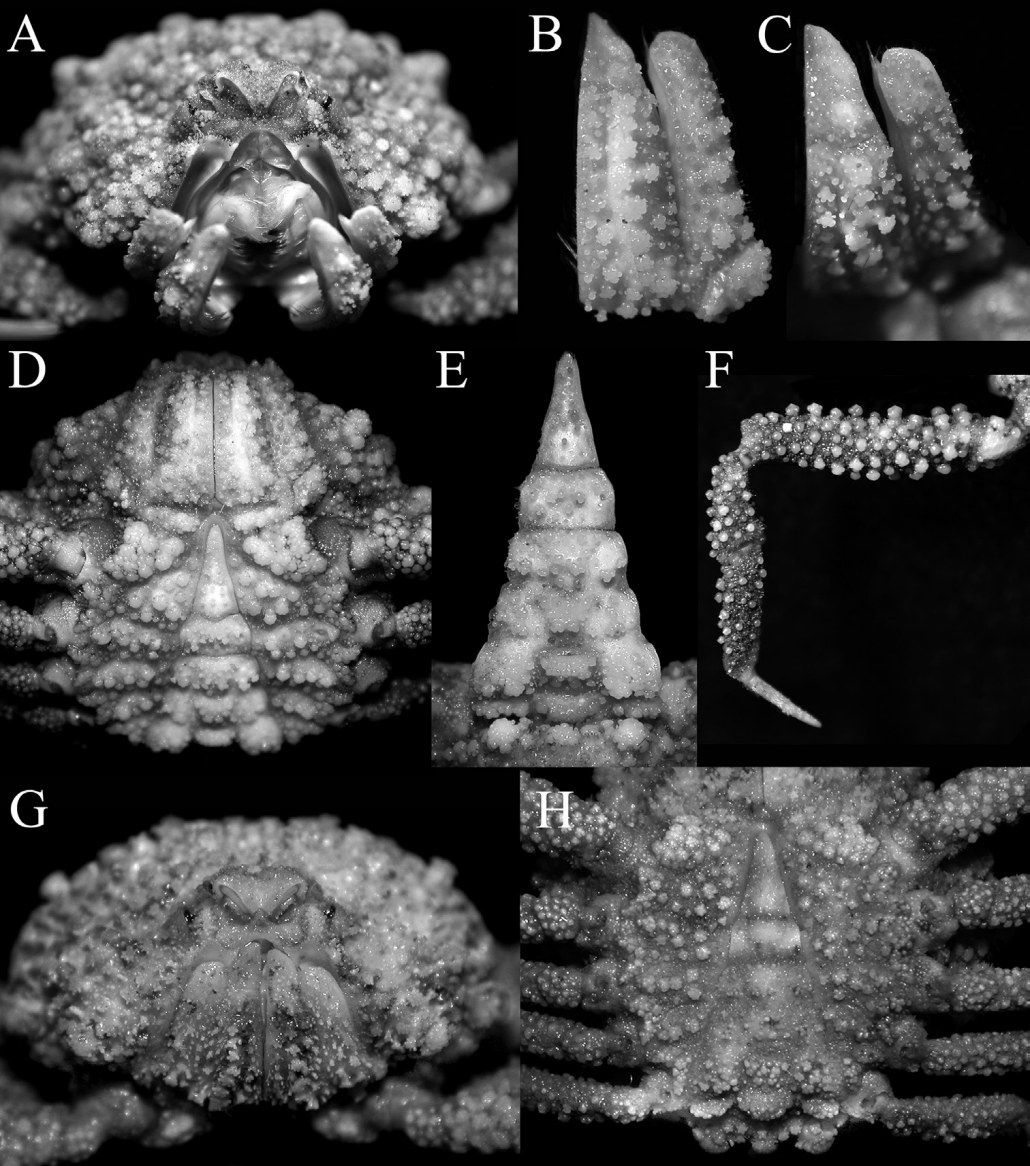
*Pariphiculus stellatus* sp. n. **A–F** holotype male (27.7 × 24.5 mm) (ASIZ 75485), Taiwan **G, H** male (24.3 × 21.6 mm) (ZRC 2007.590), Philippines. **A, G** frontal view of cephalothorax showing orbit, antennae, antennules and buccal cavity **B, C** outer view of left third maxilliped **D, H** male thoracic sternum and pleon **E** male pleon **F** left third ambulatory leg.

**Figure 9.**
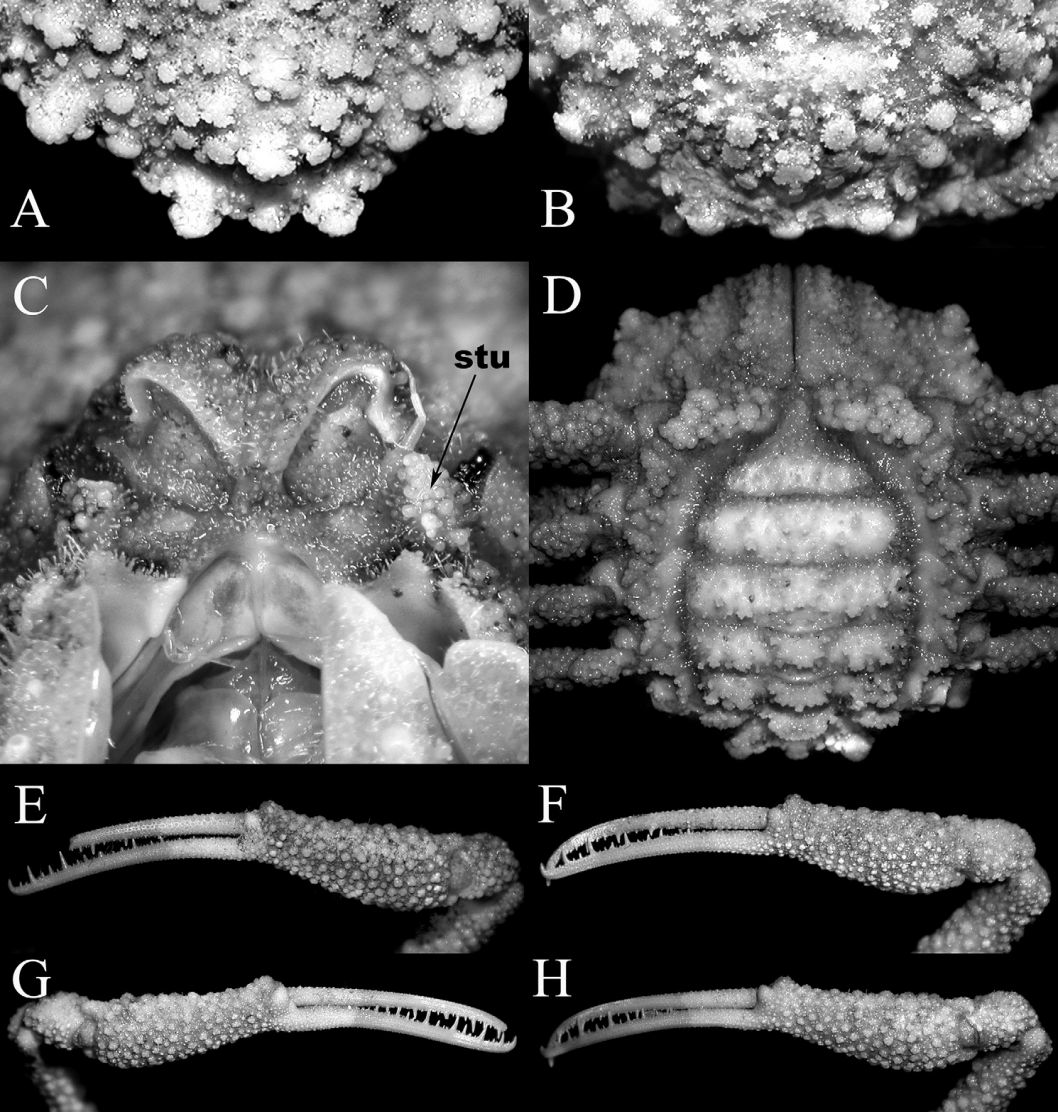
*Pariphiculus stellatus* sp. n. **A, C, E** holotype male (27.7 × 24.5 mm) (ASIZ 75485), Taiwan **B** female (32.3 × 27.6 mm) (ZRC 2017.185), Philippines **F** male (24.3 × 21.6 mm) (ZRC 2007.590), Philippines **D, G, H** female (35.1 × 29.6 mm) (ZRC 2007.590), Philippines. **A, B** intestinal region and posterior margin of carapace **C** antennae, antennules and orbit **D** female thoracic sternum and pleon **E, F, H** outer view of left chela **G** outer view of right chela. Abbreviation: stu = suborbital tubercle.

**Figure 10.**
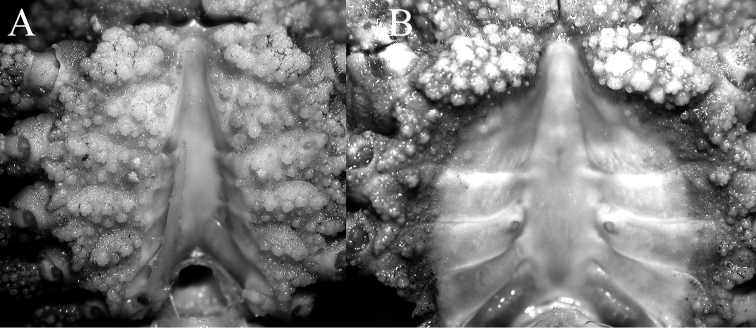
*Pariphiculus stellatus* sp. n. **A** holotype male (27.7 × 24.5 mm) (ASIZ 75485), Taiwan **B** female (31.1 × 26.2 mm) (ZRC 2017.188), Philippines. **A** male sternopleonal cavity showing right G1 and pleonal locking tubercle on sternite 5 **B** female sternopleonal cavity showing vulvae on sternite 6.

**Figure 11.**
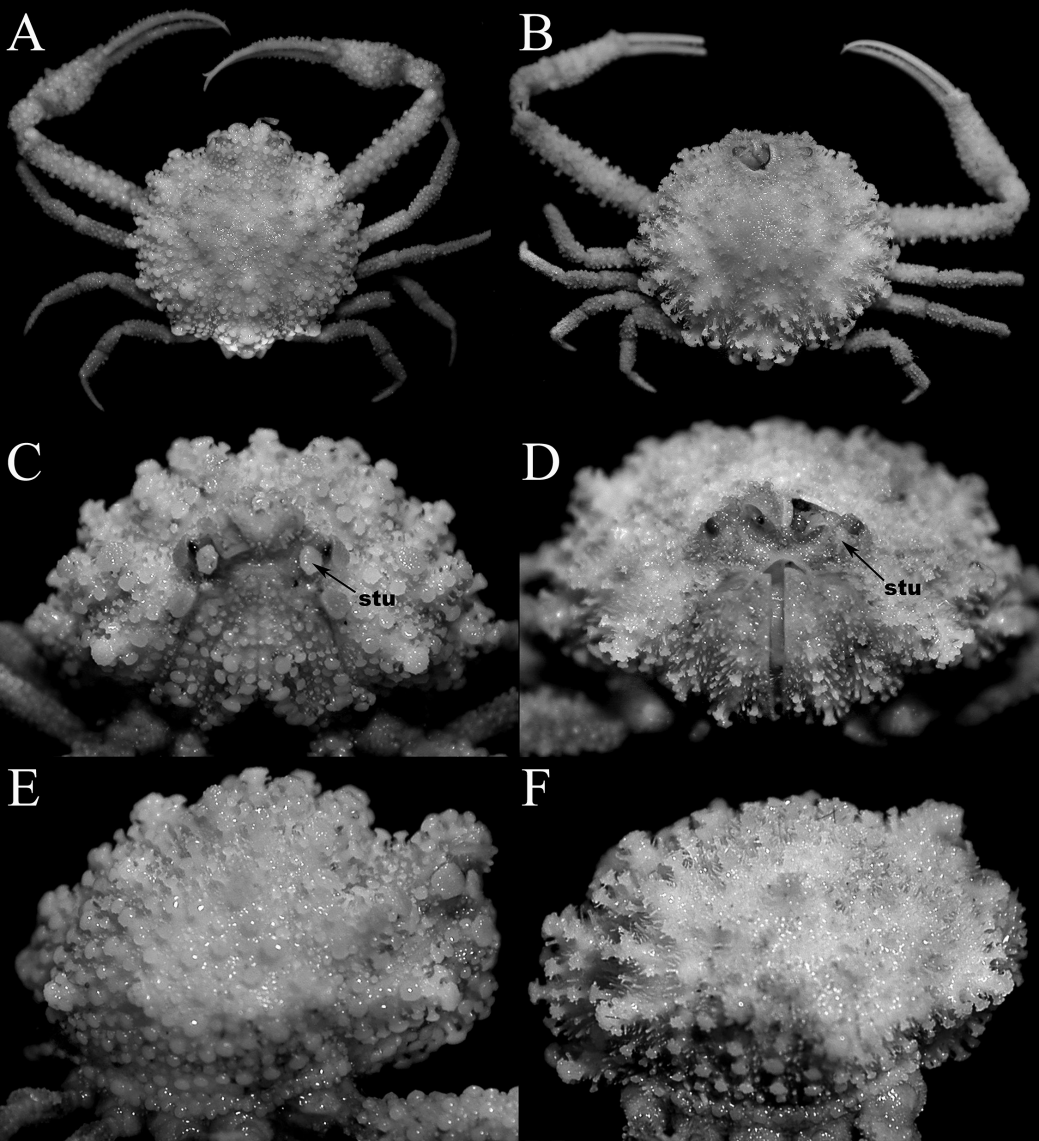
Comparisons between similar sized *Pariphiculus agariciferus* Ihle, 1918, and *P. stellatus* sp. n. **A, C, E** *P. agariciferus*, male (15.8 × 15.8 mm) (ZRC 2007.588), Philippines **B, D, F** *P. stellatus*, male (15.4 × 13.8 mm) (ZRC 2017.184), Philippines [left frontal margin damaged]. **A, B** overall dorsal view **C, D** frontal view of cephalothorax showing orbit, antennae, antennules and buccal cavity **E, F** right lateral view of cephalothorax. Abbreviation: stu = suborbital tubercle.

**Figure 12.**
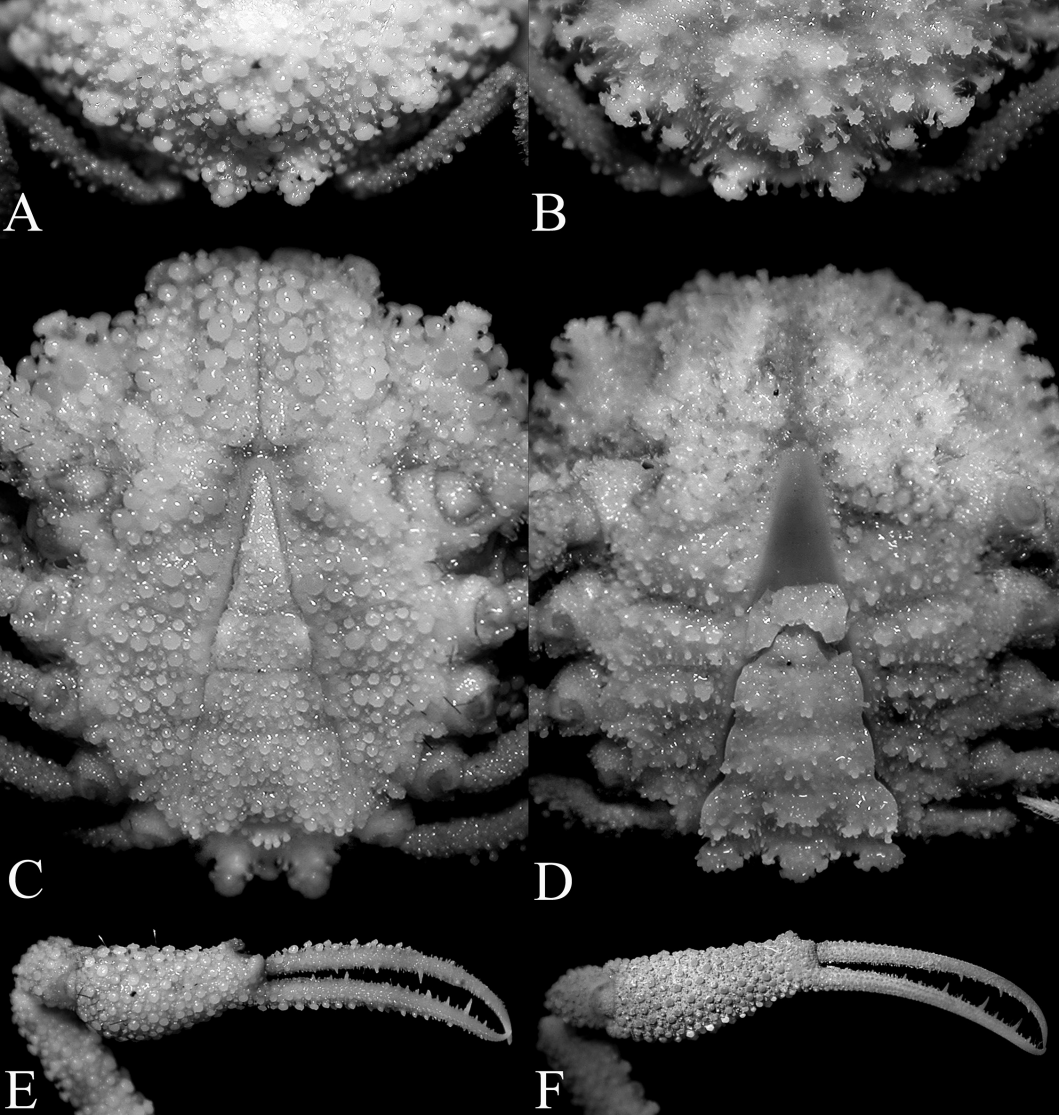
Comparisons between similar sized *Pariphiculus agariciferus* Ihle, 1918, and *P. stellatus* sp. n. **A, C, E** *P. agariciferus*, male (15.8 × 15.8 mm) (ZRC 2007.588), Philippines **B, D, F** *P. stellatus*, male (15.4 × 13.8 mm) (ZRC 2017.184), Philippines [telson missing, approximate shape and proportions as indicated by space on sternopleonal cavity]. **A, B** intestinal region and posterior margin of carapace **C, D** male thoracic sternum and pleon (somite 6 damaged and telson missing in D) **E, F** outer view of right chela.

**Figure 13.**
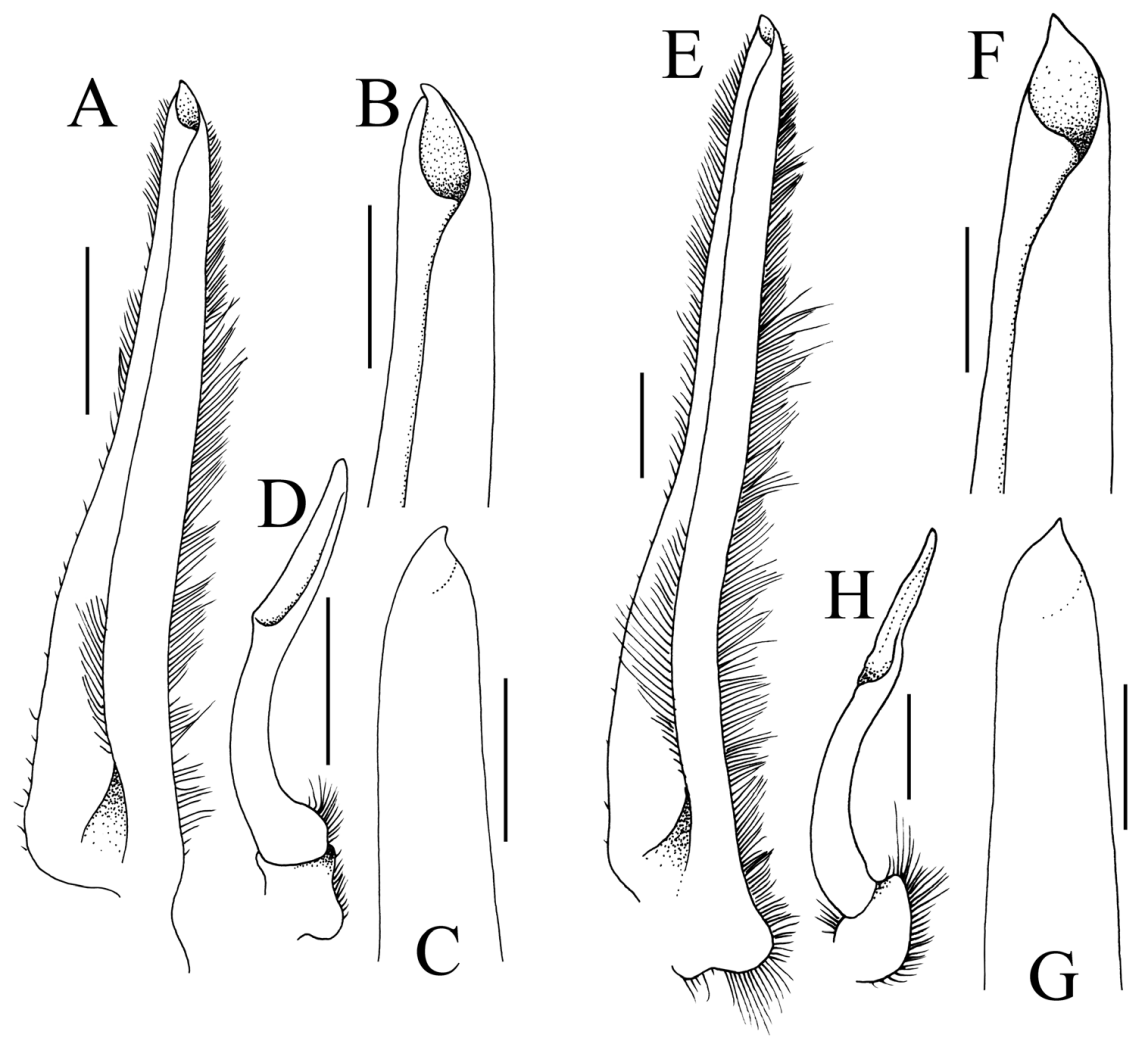
Gonopods. **A**–**D** *Pariphiculus agariciferus* Ihle, 1918, male (15.8 × 15.0 mm) (ZRC), Philippines **E–H** *P. stellatus* sp. n., holotype male (27.7 × 24.5 mm) (ASIZ 75485), Taiwan. **A–C, E–G** left G1 **D, H** left G2. Scale bars **A, E, D, H** 1.0 mm; **B, C, F, G** 0.5 mm.

Figs 7 , 8 , 9 , 10 , 11B, D, F , 12B, D, F , 13E–H

Pariphiculus sp. – Ikeda 1998: 84, pl. 2.
Pariphiculus agariciferus – Galil and Ng 2007: 87 (part) (not Pariphiculus agariciferus Ihle, 1918).

###### Material examined.

Holotype: male (27.7 × 24.5 mm) (ASIZ 75485), waters of Zone A (precious coral fishing ground), Peng-Chia-Yu Islands, 60 km northeast of Keelung city, 25°37.765'N, 122°28.623'E, 170 m, Taiwan, coll. Fishing Vessel “De-Cheng 136”, M.-L. Chang, 7 May 2017. **Philippines**: Non-types: 1 female (31.1 × 26.2 mm) (ZRC 2017.188), Balicasag Island, coll. J. Arbasto, July 2004–May 2005; 1 male (24.3 × 21.6 mm), 1 female (35.1 × 29.6 mm) (ZRC 2007.590), Balicasag Island, coll. 2 March 2004; 1 male (15.4 × 13.8 mm) (ZRC 2017.184), station PN1, Balicasag Island, coll. November 2003; 1 female (32.3 × 27.6 mm) (ZRC 2017.185), Balicasag Island, Panglao, Philippines, 80–140 m, in tangle nets, coll. March 2004 [all above locations at 9.518891°N, 123.680511°E, Panglao, Bohol, Visayas, Philippines; purchased from local shell fishermen, obtained by tangle nets].

###### Comparative material of *Pariphiculus agariciferus* Ihle, 1918.

**Taiwan**: 1 male (12.9 × 12.3 mm) (ASIZ 75113), 22°14.74'N, 118°43.69'E 141 m, southern Taiwan, coll. M.-L. Chang, 14 May 2011. **Philippines**: 1 male (16.6 × 16.2 mm) (ZRC 2015.436), Balicasag Island, coll. J. Arbasto, July 2004–May 2005; 2 females (19.5 × 18.9 mm, 19.8 × 18.9 mm) (ZRC 2017.181), Balicasag Island, coll. J. Arbasto, 2006; 1 female (19.3 × 17.6 mm), 1 broken female (ZRC 2001.559), Balicasag Island, 50–500 m, coll. 28 November 2001; 1 male (14.4 × 13.3 mm), 1 female (21.0 × 19.1 mm) (ZRC 2009.297), Balicasag Island, coll. June 2002; 2 males (13.4 × 13.3 mm, 15.8 × 15.8 mm), 1 female (19.5 × 18.4 mm) (ZRC 2007.588a), station PN1, Balicasag Island, coll. November 2003; 1 male (13.9 × 13.3 mm) (ZRC 2009.1164), Balicasag Island, coll. December 2003; 1 female (16.6 × 18.6 mm) (ZRC 2017.180), station P2, Balicasag Island, 2004; 1 male (14.0 × 13.5 mm) (ZRC 2015.437), Balicasag Island, coll. 2 March 2004; 1 male (15.8 × 15.0 mm) (ZRC 2017.187), Balicasag Island, Panglao, Philippines, 80–140 m, in tangle nets, coll. March 2004; 1 male (15.5 × 14.4 mm) (ZRC 2012.484), Balicasag Island, coll. May 2004; 1 female (22.0 × 21.6 mm) (ZRC 2017.183), station P1, Balicasag Island, coll. 6 July 2004; 1 young female (15.0 × 14.7 mm) (ZRC 2017.182), station P4, Balicasag Island, coll. 2 July 2004; 2 females (18.4 × 18.0 mm, 22.1 × 21.3 mm) (ZRC 2009.288), Balicasag Island, coll. J. Arbasto, July 2004–May 2005; 1 male (12.8 × 13.7 mm), 1 female (19.0 × 19.0 mm) (ZRC 2017.186), Balicasag Island, Panglao, Philippines, 80–140 m, in tangle nets, coll. J. Arbasto, 2004–2005; 1 male (15.4 × 16.0 mm) (ZRC 2009.185), Balicasag Island, 120–160 m, coll. J. Arbasto, January–December 2007 [all above locations at 9.518891°N, 123.680511°E, Panglao, Bohol, Visayas, Philippines; purchased from local shell fishermen, obtained by tangle nets]; 1 male (17.2 × 16.9 mm), 2 females (16.1 × 15.4 mm, 18.5 × 18.7 mm) (ZRC 2013.104), Maribojoc Bay, Panglao, Bohol, Philippines, coll. J. Arbasto, November 2003–April 2004. **Vanuatu**: 1 male (10.2 × 9.6 mm), 1 female (13.0 × 13.5 mm) (ZRC 2009.470), station AT13, south of Aésé Island, Palikulu Bay, 15°27.8S 167°15.7E, 146–153 m, Santo, coll. N.O.”Alis”, SANTO Expedition, 19 September 2006; 1 male (11.9 × 12.1 mm) (ZRC 2009.471), station AT61, south of Urélapa Island, West Malo Island, 15°39'S 167°01'E, 266–281 m, Santo, coll. N.O.”Alis”, SANTO Expedition, 4 October 2006; 2 juvenile females (10.3 × 9.4 mm, 13.4 × 13.6 mm), 1 carapace only (17.9 × 17.4 mm) (ZRC 2009.472), station AT63, south of Urélapa Island, West Malo Island, 15°40'S 167°01'E, 290–334 m, Santo, coll. N.O.”Alis”, SANTO Expedition, 4 October 2006; 1 male (11.9 × 11.8 mm) (ZRC 2009.473), station AT64, south of Urélapa Island, West Malo Island, 15°40'S 167°02'E, 249–252 m, Santo, coll. N.O.”Alis”, SANTO Expedition, 19 September 2006.

###### Diagnosis.

Carapace 1.12–1.19 times broader than long; with relatively low mushroom-shaped tubercles on carapace, chelipeds and ambulatory legs with margins of tops distinctly asteriform (Figs 7, 8A, G, 9A, B, 11D, F, 12B); gastric and branchial regions inflated (Figs 8A, G, 11D, F); in lateral view, surface behind postfrontal region gently concave, forming shallow depression (Fig. 11F); suborbital tubercle relatively low, not protruding (Figs 8A, G, 9C, 11D); palms of chelae relatively long, slender, with fingers long, gently curved (Figs 9E–H, 12F); dorsal margin of dactylus and ventral margin of pollex of chela lined with low granules, never serrated (Figs 9E–H, 12F); tubercles and granules on surface of male and female pleons relatively large, tend to be fused, forming semi-eroded structures (Figs 8D, E, 9D, 12D); male telson proportionately shorter (Figs 8D, E, 12D); G1 relatively long, slender (Fig. 13E).

###### Description of male.

*Carapace* 1.12–1.19 times broader than long, regions not well-defined; dorsal surface with numerous low mushroom-shaped tubercles and granules, edges of raised tubercle with short slender spinules, asteriform from dorsal view, with scattered short setae between them; postfrontal region raised, prominent; surface behind postfrontal region with shallow depression; gastric regions convex, separated from swollen branchial regions by shallow, partially granulated groove; branchial regions with numerous large and small tubercles, separated from intestinal and cardiac regions by relatively broad granulated groove; cardiac and intestinal regions barely distinguishable, intestinal region with 2 prominent large tubercles, one dorsal in position, another directed obliquely posteriorly; hepatic region gently concave, with distinct low lateral tubercle, surface granulated; pterygostomial and suborbital regions with numerous mushroom-shaped, asteriform tubercles; branchiostegite region with numerous low, rounded tubercles (Figs 7, 8A, G, 9A–C, 11B, D–F, 12B). posterolateral border adjacent to cardiac region prominently protruded to form large tooth. Front slightly produced, not protruding beyond anterior edge of buccal cavity and closed third maxillipeds, gently upturned, weakly bilobed (distinct in frontal view) (Figs 7, 8A, G, 11B, D, 12B). Antero- and posterolateral margins not cristate, not clearly demarcated, with structures gradually merging medially on carapace; subdorsal margins lined with numerous large mushroom-shaped tubercles with asteriform tops, with interspersed smaller granules of similar form; posterior carapace margin with 3 broad, stout tubercles, each covered with smaller granules, median one smallest (Figs 7, 9A, B, 11B, 12B).

Basal article of *antenna* subquadrate, surface gently convex, fused with epistome; short flagellum lodged in orbital hiatus (Fig. 9C). Basal segment of antennule occupies entire fossa when closed, with folded articles hidden behind basal article; margins of fossae almost smooth (Figs 8A, G, 9C). Orbit rounded; eyes small with short ocular peduncle, mobile; suborbital tubercle relatively low, not prominent, asteriform (Figs 8A, G, 9C, 11D).

*Third maxillipeds* relatively short; outer surfaces of merus, ischium, basis and exopod densely covered with mushroom-shaped tubercles and granules, many with asteriform tops; merus triangular with broadly pointed apex, inner edge straight, outer edge gently convex, with large, low rounded tubercle on proximal margin; ischium ca. 2 times longer than merus along inner margin, with prominent submedian ridge of tubercles; palp (carpus, propodus and dactylus, short, completely hidden behind ischium when folded; exopod relatively broad, reaching beyond distal margin of ischium, tip rounded, without flagellum (Figs 8B, C, G 11D).

*Chelipeds* slender, elongate, subequal; surfaces with numerous large, round or low mushroom-shaped tubercles, some with asteriform tops (Figs 7A, F, G, 9E–H, 11B, 12F). Merus cylindrical in cross-section; carpus small, subtriangular (Figs 7A, F, G, 9E–H, 11B). Chela with proximal half relatively stouter, gradually tapering to more slender distal part, margins granulated but not serrated; fingers elongate, just longer than palm, surface with very small granules, gently curved, distal two-thirds more darkly pigmented, rest of structure white in preservative; cutting edges of both fingers with long and short sharp vertical spines (Figs 9E–H, 12F).

*Ambulatory legs* relatively short, first leg longest; surfaces of merus, carpus and propodus covered with small rounded or mushroom-shaped tubercles, some with asteriform tops (Figs 7A, F, G, 8F, 11B); dactylus styliform, almost straight, surfaces smooth, margins lined with setae (Fig. 8F).

*Thoracic sternum* relatively narrow transversely; surfaces of sternites 1–4 covered with round or low mushroom-shaped tubercles, some with asteriform tops, some coalescing to form ridges and clusters; surfaces of sternites 5–8 with more individual tubercles and granules; sternites 1 and 2 separated by low ridge, longitudinally very narrow, lateral surfaces of sternite 2 with thick row of coalesced tubercles; separated from coalesced row of tubercles on sternite 3 by deep groove; tubercles on sternite 3 separated from cluster of coalesced tubercles on sternite 4 by deep groove; sternites otherwise not clearly demarcated; small part of sternite 8 just visible when pleon closed; sternopleonal cavity narrow, deep, nearly reaching buccal cavity at level of sternite 2 (Figs 8D, H, 12D); all sutures from sternites 3–8 medially interrupted; peg-like tubercle of pleonal locking mechanism relatively large, semicircular, directed anteriorly (Fig. 10A); penis short, tubular with dilated tip, arising from condyle of coxa of fourth ambulatory leg (Fig. 10A).

*Pleon* triangular, covered with large rounded tubercles, many coalescing to form semi-eroded structure on somites 1–6; surface of telson with scattered small, narrow mushroom-shaped; somites 1, 2 free; somites 3–5 functionally fused although somites can still be approximately distinguished; somites 2–6 trapezoidal; somite 6 free, broadly subrectangular with lateral margin convex; telson triangular, elongate; surface of somite 3 with subrectangular cluster of fused granules; surface of somite 2 with 3 uneven clusters of fused granules (Figs 8D, E, H, 12D).

*G1* ca. 2 times length of G2, relatively slender, slightly sinuous basally, becoming almost straight distally; margins lined with dense soft long setae; tip sharp (Fig. 13E–G). G2 with with elongate subpetaloid terminal process which is slightly shorter than basal segment (Fig. 13H).

###### Females and variations.

Female specimens are similar to males in almost all non-sexual aspects. The female pleon is of the typical iphiculid condition, with all the somites and telson freely articulating, none swollen or forming a dome-like structure covering the egg mass (Fig. 9D). The vulva is relatively small and simple with around opening, covered by an operculum, and directed obliquely posteriorly (Fig. 10B). Small specimens have proportionately shorter meri of the chelipeds although in all other aspects, the structures are similar (Figs 7B, F, 11B).

###### Colour.

The fresh holotype of *P. stellatus* was a dull orange throughout, with some of the large posterior tubercles white; the distal two-thirds of fingers bright orange and basal third cream (Fig. 1E). The ventral surfaces are generally dirty white to light brown (Fig. 1F). A relatively larger fresh specimen of *P. agariciferus* (18. 1 mm carapace width) was figured by Galil and Ng (2007: fig. 3B) and its colour was similar to that of *P. stellatus*, being orange throughout. A smaller male specimen from Taiwan (12.9 × 12.3 mm, ASIZ 75113), however, had most of the carapace and chelipeds white and pale orange (Shih et al. in press). A similar sized male from Vanuatu (11.9 × 12.1 mm, ZRC 2009.471) had a similar colour pattern as the Taiwan specimen except that the colour is more intense (Fig. 1D)

###### Etymology.

The species is named after the prominent asteriform or “star-like” mushroom-shaped tubercles and granules on the carapace and chelipeds.

###### Remarks.

The large specimen from Taiwan is interesting as it is almost identical with a large female specimen measuring 36.2 × 30.3 mm figured by Ikeda (1998: 84) from Sagami Bay which he identified as “*Pariphiculus* sp.”. Ng (1999: 718), in his review of the book, noted that this was almost certainly a new species although he incorrectly suggested it may be a species of *Parilia* Wood-Mason in Wood-Mason & Alcock, 1891, instead. Examination of the present Taiwanese specimen suggests it is close to *Pariphiculus agariciferus* Ihle, 1918, a species known from Indonesia, Japan, Philippines, Taiwan, South China Sea and Vanuatu (Ihle 1918: 250; Balss 1922: 131; Yokoya 1933: 129; Sakai 1937: 131; Sakai 1965: 43; Serène and Vadon 1981:124; Sakai 1976: 104; Chen 1989: 231; Tan 1996: 1023; Komatsu et al. 2005: 106; Galil and Ng 2007: 87; Galil and Ng 2010: 143; Ng et al. 2008: 37; Shih et al. in press). The size of the Taiwanese (and Ikeda’s Japanese) specimen, however, is much larger (almost twice) that what is known for *P. agariciferus* which averages 15–20 mm in carapace width. The Taiwanese specimen differs from typical *P. agariciferus* in a number of carapace, cheliped, male pleonal and G1 characters that suggests that it is a different taxon, but its large size makes comparisons difficult.

Fortunately, there is a good series of specimens of what had been identified as “*P. agariciferus*” from Philippines and Vanuatu by Galil and Ng (2007, 2010), as well as additional material in the ZRC not reported by them. While the majority of the specimens are *P. agariciferus* s. str., there are several specimens mixed among this material (cf. Galil and Ng 2007) that are clearly conspecific with the Taiwan specimen. This includes a small male specimen of the new species which allows for size-equivalent comparisons between the two taxa to be made. We can now be confident that the present Taiwanese male, Ikeda’s (1998) specimen, and some of the Philippine material represent a new species, here named *Pariphiculus stellatus* sp. n.

*Pariphiculus agariciferus* Ihle, 1918, is a smaller species, with all specimens less than 22 mm in carapace length. The holotype male from seas around Timor and Rotti islands in Indonesia measured 9.0 × 9.3 mm (not measured to tip of spines). The carapace of the holotype male has all the lateral spines relatively slender and long, all of which are covered with additional spinules and tubercles; with the tubercles and granules on the carapace and chelipeds mushroom-shaped (Fig. 4A). A slightly larger male from Vanuatu has a very similar in carapace morphology (Fig. 4B). These characters are almost certainly juvenile features; the small Vanuatu specimen is still subadult, with the gonopods poorly chitinised; and the holotype male is probably a young individual as well. In the larger male specimens from the Philippines, Taiwan and Vanuatu, the lateral spines (including those on the posterior carapace margin) are relatively shorter and stouter (Figs 4C–F, 6A–F). The ambulatory meri and dactyli of the smallest specimens are also proportionately more slender and elongate (Fig. 4A, B). As specimens get larger (regardless of sex), the carapace becomes proportionately wider and rounder, and the spines shorter and blunter (Fig. 4C–F). The ambulatory meri and dactyli of these larger specimens are also relatively stouter and shorter (Fig. 4C–F). As the specimens increase in size, the tops of the mushroom-shaped tubercles on the carapace and chelae also become more mushroom-shaped, with the margins more gently serrated and distinctly asteriform (Figs 4C–F, 6A–F).

*Pariphiculus stellatus* sp. n. can be distinguished from *P. agariciferus* in having the gastric and branchial regions of the carapace proportionately less inflated (Figs 8A, G, 11D, F) (vs. prominently inflated in frontal view in *P. agariciferus*; cf. Fig. 11C, E); the tubercles on the carapace, chelipeds and third maxillipeds are prominently mushroom-shaped but relatively lower, with the tops of the large tubercles distinctly asteriform (Figs 8A–C, G, 9A, B, 11D, F, 12B) (vs. tubercles on the carapace are relatively higher and mushroom-shaped, with the margins of the tops of the tubercles uneven but not distinctly asteriform in *P. agariciferus*; cf. Figs 5A, B, 6A–F, 11C, E, 12A); in lateral view, the surface behind the postfrontal region is gently concave, forming a shallow depression (Fig. 11F) (vs. area behind postfrontal region deeply concave, with a marked depression between frontal and gastric regions in *P. agariciferus*; cf. Fig. 11E); the suborbital tubercle is relatively low, not protruding and not conspicuous (Figs 8A, G, 9C, 11D) (vs. suborbital tubercle is large and prominently protrudes anteriorly in *P. agariciferus*; cf. Fig. 11C); the palms of the chelae are relatively long, slender with the fingers gently curved (Figs 9E–H, 12F) (vs. palms relatively short, swollen with the fingers distinctly curved in *P. agariciferus*; cf. Figs 6G–J, 12C); the dorsal margin of the dactylus and ventral margin of the pollex of the chela is lined with low granules, never serrated (Figs 9E–H, 12F) (vs. non-cutting margins of dactylus and pollex with prominent sharp and/or mushroom-shaped granules, appears serrated in *P. agariciferus*; cf. Figs 6G–J, 12C); the tubercles and granules on the surface of the male and female pleons are larger, tend to be fused, forming semi-eroded structures (Figs 8D, E, H, 9D, 12D) (vs. tubercles and granules tend to be smaller, discrete and packed in *P. agariciferus*; cf. Figs 5C–H, 12C); the male telson is proportionately shorter, even in small specimens (Figs 8D, E, H, 12D) (vs. male telson more elongate in *P. agariciferus*; cf. Figs 5C–F, 12C); and the G1 is relatively longer and more slender (Fig. 13E) (vs. G1 proportionately stouter and shorter in *P. agariciferus*; cf. Fig. 13A). It is important to note also that the single male specimen of *P. stellatus* sp. n. (Figs 11B–F, 12B–F) similar in size to adult *P. agariciferus* (Figs 11A, C, E, 12A, C, E) is still subadult, with the G1 soft and not well developed.

All the specimens of *P. stellatus* sp. n. have been collected by tangle nets in Taiwan and the Philippines. This is also true for all the *P. agariciferus* collected from the Philippines, with only a few specimens obtained by trawls (e.g. those in Vanuatu and Taiwan). The material from the Philippines was collected from deeper waters along steep cliffs, areas which can neither be sampled by divers, trawls or dredges (Ng et al. 2009). These heretofore rare species almost certainly prefer such inaccessible habitats and are thus rarely collected by dredges and trawls (see Mendoza et al. 2010).

###### Distribution and depth.

Ikeda’s (1998) Japanese specimen was collected from 180–200 m of water off Nagai in Sagami Bay. The holotype male from Taiwan was obtained from a depth of 170 m, with the series from the Philippines collected from 80–140 m.

## Supplementary Material

XML Treatment for Acanthodromia margarita

XML Treatment for Pariphiculus stellatus
